# Rapamycin is Effective for Upper but not for Lower Gastrointestinal Crohn’s Disease-Related Stricture: A Pilot Study

**DOI:** 10.3389/fphar.2020.617535

**Published:** 2021-02-04

**Authors:** Min Zhong, Bota Cui, Jie Xiang, Xia Wu, Quan Wen, Qianqian Li, Faming Zhang

**Affiliations:** ^1^ Medical Center for Digestive Diseases, The Second Affiliated Hospital of Nanjing Medical University, Nanjing, China; ^2^ Key Lab of Holistic Integrative Enterology, Nanjing Medical University, Nanjing, China; ^3^ Department of Gastroenterology, The Central Hospital of Enshi Autonomous Prefecture, Enshi, China

**Keywords:** Crohn’s disease, rapamycin, fibrosis, stricture, inflammatory bowel diseases, duodenum obstruction

## Abstract

Crohn’s disease (CD)-related fibrotic stricture remains a clinical challenge because of no effective treatments. This study aimed to evaluate the potential efficacy of rapamycin in patients with CD-related strictures in different locations in gastrointestinal tract. A pilot prospective study on using rapamycin for CD-related stricture was performed from April 2015 to August 2020 in a single center in China. Fifteen patients were enrolled into the study. The clinical efficacy was evaluated by diet score and gastrointestinal obstruction symptoms score. Clinical responses were defined as the ability to tolerate the regular diet with vegetable fiber combined with a reduction of ≥75% in overall target score and a score of less than two points for each item. Three patients discontinued rapamycin for less than 1-month due to intolerance to adverse events, then, 12 patients received ≥1 dose of the rapamycin and provided ≥1 post-baseline target score after baseline were included for intent-to-treat (ITT) analysis. 100% (5/5) of patients with upper gastrointestinal strictures achieved clinical response after using rapamycin. However, no clinical response was observed in those patients with CD lesions in lower gastrointestinal tract. Adverse events occurred in 40% (6/15) of patients. No death or serious opportunistic infections were observed in the present study. This study firstly reported that rapamycin might be effective for CD-related stricture in the upper, but not in lower gastrointestinal tract.

## Introduction

Crohn’s disease (CD) is a chronic relapsing inflammatory disease that can occur in any segment of the gastrointestinal tract. However, the naturally progressive disease course culminates in stricture formation (Cosnes et al., 2011), often leading to repeated bowel obstruction and surgery (Rieder et al., 2013; Singh et al., 2017). Mostly, strictures are caused by the combination of inflammation and fibrosis, and the intensity of fibrosis is almost impossible to determine (Feakins, 2020). Clinically, CD-related stenosis can be silent or symptomatic. Symptomatic stenosis may manifest as postprandial bloating, or significant intestinal obstruction, causing nausea, vomiting, and abdominal pain. Up till now, the therapy of choice for CD with fibrotic strictures, mainly comprises endoscopic dilation endoscopic stricturotomy and surgery (Kanazawa et al., 2012; Singh et al., 2017; Shen et al., 2020), in conjunction with purely anti-inflammatory therapy. Although endoscopic dilation procedures for stricturing CD are usually technically successful, the majority of patients still required multiple sessions of endoscopic dilation, and some might develop perforation (Kanazawa et al., 2012; Singh et al., 2017). A significant number of patients have to undergo multiple surgeries, with the attendant risk of developing short bowel syndrome and intestinal failure (Rieder et al., 2013). Therefore, there is necessity to explore more effective treatments for patients with CD-related fibrotic stricture.

In recent studies, rapamycin, a serine/THR kinase inhibitor of mammalian target (mTOR), has been reported as potentially effective treatment in limited populations with refractory CD (Massey et al., 2008; Mutalib et al., 2014). Rapamycin has also been reported to inhibit the progression of kidney fibrosis (Chen et al., 2012), cardiac fibrosis (Haller et al., 2016), and pulmonary fibrosis (Xu et al., 2017). A recent study has shown that rapamycin can reduce intestinal fibrosis by inhibiting CX3Cr1+ mTOR/autophagy in mononuclear phagocytes and up-regulating the IL-23/IL-22 axis (Mathur et al., 2019). Based on the consideration on the two facts: the very low incidence of CD patients with upper gastrointestinal fibrotic stricture (Nugent et al., 1989; Van Assche et al., 2004), and the hypothesis on the local anti-fibrosis effect of rapamycin in upper section of small intestine, the present study aimed to evaluate the potential efficacy of rapamycin for patients with CD-related gastrointestinal stricture.

## Materials and Methods

### Study Design and Participants

Patients with CD from the nation came to the Second Affiliated Hospital of Nanjing Medical University for seeking fecal microbiota transplantation based on automatic filtration and washing process and the related delivery, which was called as washed microbiota transplantation (WMT) (Fecal Microbiota Transplantation-standardization Study Group, 2020; Zhang et al., 2020). However, these patients with CD-related strictures were not considered for WMT, but invited to attend the present trial (NCT02675153). The study was approved by the Second Affiliated Hospital of the Nanjing Medical University Institutional Ethical Review Board (2016KY001), and written informed consent was obtained from all patients. Demographics (age, gender) and disease characteristics (disease location, duration, duration of stricture and other CD drug medications) of each patient were noted before the study.

### Inclusion and Exclusion Criteria

Inclusion criteria were: (1) Patients (≥18 years of age) with a documented definite diagnosis of CD; and 2) the presence of a clinically symptomatic stricture; and 3) strictures confirmed by endoscopy (passing endoscope with difficulty) or typical image by CT enterography (CTE) or MR enterography (MRE). Patients were excluded: 1) patients who were pregnant, diagnosed with intestinal perforation, complete intestinal obstruction, any signs of dysplasia or malignancy, or use of anti-tumor necrosis factor (TNF) in the last three months; and 2) patients who were not followed up between the inception of medication and any other subsequent treatments.

### Procedures and Outcome Measures

Patients were treated with rapamycin (2 mg/day, Sirolimus, Roche) and short-term enteral nutrition. Patients with colitis were also treated with 5-aminosalicylic acid (5-ASA). The intent-to-treat (ITT) population comprised all patients who received ≥1 dose of the study drug and provided ≥1 post-baseline target score after baseline.

We used a composite score considering all the essential elements including the gastrointestinal obstruction symptoms score and diet score to assess the effectiveness of treatment in clinical practice during follow-up. Response should meet the following three criteria: (a) the ability to tolerate a normal diet (vegetable fiber), with a reduction of ≥75% in overall baseline target score and sub-score ≤ 2 (Table 1); (b) no need for endoscopic dilation or surgery; (c) no severe adverse events or any other reasons leading to rapamycin withdrawal. The primary aim was the rate of clinical response focusing on obstruction after using rapamycin. The follow-up was at least 6 months for patients with unclear efficacy. Patients who prematurely discontinued rapamycin due to severe adverse events or who did not meet clinical response criteria after 6 months medication usage were considered treatment failure. The secondary aim was to evaluate the adverse events during medication usage in all enrolled patients. In this study, new onset of symptoms and the exacerbation of previous symptoms were recorded as adverse events. We also evaluated the primary endpoint was the rate of surgery or ED after rapamycin as the long-term treatment outcomes.

**TABLE 1:** Definition of each target and scoring method in patients.

Variables	Scoring method
Abdominal pain	0: No abdominal pain
	1–2: Essentially normal
	3–5: Affecting daily life but not sleeping
	6–9: Affecting sleep
Abdominal distention	0: No abdominal distention
	1–2: Essentially normal
	3–5: Affecting daily life but not sleeping
	6–9: Affecting sleep
Diet	0: No dietary restrictions
	1–2: Essentially normal, without nausea and vomiting
	3–5: Regular diet, with nausea and vomiting
	6–8: Fluid diet and enteral nutrition, with nausea and vomiting
	9: Exclusive enteral nutrition or gastrointestinal decompression

### Statistical Analysis

Data were analyzed using IBM SPSS Statistics 23.0.0 (SPSS Inc., Chicago, IL, United States). Continuous variables were expressed using median and interquartile range and tested by Analysis of Variance test. Categorical data were described as number (percentages) and were tested by Chi-square analysis or Fisher’s exact test. The paired data were compared using the Paired *t* test. *p* < 0.05 was indicative of statistical significance.

## Results

From April 2015 to August 2020, totally 15 patients were enrolled. The follow-up finished on December 1, 2020. The patient demographics, characteristics of CD, and previous history of drug therapy at the baseline were well balanced between the treatment groups (as shown in Table 2) and three of them with lesions in ileum or colon were administered oral 5-ASA simultaneously. One patient in the upper gastrointestinal group and two patients in the lower gastrointestinal group stopped to take the medication within one month because of adverse events (Table 2) and did not provide a post-baseline target score. Therefore, 15 patients were included in the safety population and 12 patients were included for in the ITT population (Table 3).

**TABLE 2:** Characteristics of the patients at baseline.

	All patients (n = 15)	Upper gastrointestinal (n = 6)	Lower gastrointestinal (n = 9)	*p* value
Age, years, median (IQR)	30 (28.5–38.5)	29 (24.3–30.8)	31 (29–39)	0.358
Male, n (%)	8 (53.3%)	4 (66.7%)	4 (44.4%)	0.608
Family history of CD, n (%)	0 (0.0%)	0 (0.0%)	0 (0.0%)	
Smoking, n (%)				0.604
Current	0 (0.0%)	0 (0.0%)	0 (0.0%)	
Ex-smoker	4 (26.7%)	1 (16.7%)	3 (33.3%)	
Never	8 (73.3%)	5 (83.3%)	6 (66.7%)	
Age at diagnosis, years, median (IQR)	25 (23–29.5)	24 (21.5–28.8)	26 (23–29)	0.852
Months from CD diagnosis to confirm the stricture, months, median (IQR)	35 (13–49)	7 (1.3–21)	46 (35–68)	0.367
Disease duration, years, median (IQR)				
	9 (4.5–12)	3 (3–7)	12 (10.5–12.5)	0.082
Fistulizing disease, n (%)				
	5 (33.3%)	2 (33.3%)	3 (33.3%)	
History of gastrointestinal surgery, n (%)				
	3 (20%)	0 (0.0%)	3 (33.3%)	
History of medications, n (%)				
				0.556
5-ASA	9 (60%)	3 (50%)	6 (66.7%)	
PPI	2 (13.3%)	2 (33.3%)	0 (0.0%)	
Immunosuppressants	9 (60%)	3e (50%)	6 (66.7%)	
Corticosteroids	8 (53.3%)	4 (66.7%)	4 (44.4%)	
Biological therapy				
	2 (13.3%)	1 (16.7%)	1 (11.1%)	
Adverse events				0.091
Palpation	2 (13.3%)	0 (0.0%)	2 (22.2%)	
Loss of libido	2 (13.3%)	0 (0.0%)	2 (22.2%)	
Abnormal liver enzymes	1 (6.7%)	0 (0.0%)	1 (11.1%)	
Mouth ulcer	2 (13.3%)	0 (0.0%)	2 (22.2%)	
Headache	1 (6.7%)	0 (0.0%)	1 (11.1%)	
Rash	0 (0.0%)	1 (16.7%)	2 (22.2%)	
Leukopenia	1 (6.7%)	1 (16.7%)	0 (0.0%)	

CD, Crohn’s disease; 5-ASA, 5-aminosalicylic acid; PPI, proton pump inhibitors; IQR, inter quartile range.

**TABLE 3:** Medications and clinical outcomes of patients.

Pt	Classification	5-ASA	Rapa duration (months)	Response (months)	HBI		Target score					
					Before	After^a^	Abdominal pain		Abdominal distention		Diet	
							Before	After^a^	Before	After^a^	Before	After^a^
1	A1, L3L4, B2	No	24	Yes (2)	2	0	0	0	5	0	5	2
2	A2, L3L4, B2	Yes	6	Yes (5)	3	0	3	0	5	0	5	0
3	A1, L4, B2	No	24	Yes (5)	2	0	0	0	0	0	6	0
4	A2, L4, B2	No	10	Yes (6)	5	1	6	0	2	0	9	1
5	A2, L4, B2	No	4	Yes (4)	3	0	0	0	5	2	9	3
6	A2, L3, B2	No	12	No	5	1	5	4	2	2	5	5
7	A2, L3, B2	Yes	11	No	5	3	5	2	1	1	6	6
8	A2, L3, B2	No	6	No	5	4	6	5	5	4	6	6
9	A2, L3, B2	Yes	6	No	5	3	3	3	3	3	3	3
10	A2, L2, B2	No	8	No	3	2	0	0	3	2	3	3
11	A2, L2, B2	No	6	No	2	2	0	0	2	2	3	3
12	A2, L3, B2	No	6	No	10	8	6	4	0	0	3	3

Rapa, Rapamycin; HBI, Harvey-Bradshaw Index; A1, ≤16 years; A2, 17–40 years; L2, colonic; L3, ileocolonic; L4, isolated upper gastrointestinal; B2, structuring; 5-ASA, 5-aminosalicylic acid.

^a^ The time that patients achieved response or received rapamycin for 6 months.

Response rate was 41.7% (5/12) in this study. The patients achieved response were all patients with upper gastrointestinal stricture (lesions in stomach and duodenum), but clinical failure was confirmed in all patients with lower lesions. 100% (5/5) of patients with upper gastrointestinal strictures achieved clinical response within 6 months after using rapamycin, with the mean time of 4.4 months. The overall target score of the patients decreased from the mean of 12.0 at baseline to 1.6 at 4.4 months (Paired *t* test*, p* = 0.005), with each target ≤2. By contrast, the mean score for patients with lower gastrointestinal lesions dropped to 8.7 at 6 months from 10.0 at baseline (Paired *t* test*, p* = 0.022). The adverse events as the secondary aim of the present study occurred in six patients (6/15, 40%) (Table 2). No death or serious opportunistic infections were reported.

The median duration of rapamycin in patients with upper gastrointestinal stenosis was 17 (Interquartile range (IQR): 9–24) months. At a median follow-up of 49 (IQR: 42–53.5) months, three of these patients were identified to be in long-term success and have been able to completely cease medication for more than one year without relapse. One case reported clinical improvement on her obstruction in upper gastrointestinal tract after 6 months of rapamycin, but she developed rectovaginal fistula after the cessation of rapamycin for 16 months. Rapamycin was discontinued in four patients with lower gastrointestinal tract stenosis after 6 months, and three of them continued to receive rapamycin for several months of their own volition, with a median duration of 6 (IQR: 6.0–9.5) months. The median time to follow-up was 37 (IQR: 29–46) months in the lower lesions group. One patient underwent surgery 17 months after rapamycin withdrawal, while the others were treated with other immunosuppressive agents after failure of rapamycin treatment.

## Discussion

Treatment of CD-related stricture is a clinical challenge, especially the lesions located in upper gastrointestinal tract, and is also a high socio-economic burden due to frequent hospitalizations and surgery (Bodger et al., 2009). Despite advances in anti-inflammatory therapies in recent decades, the incidence of intestinal stenosis in CD has not changed significantly. Inflammation and fibrosis are associated, in most cases, but to different degrees. Although surgery is the choice for fibrotic stenosis, post-operative disease recurrence and re-stricture are common (Rieder et al., 2013). Endoscopic dilatation is relatively safe for patients, but the efficacy is of limited duration (Hassan et al., 2007; Taida et al., 2018). Given the current uncertainties of the medical treatment regarding the formation and progression of fibrotic strictures, new treatments are welcome.

The efficacy of mTOR inhibitors in the treatment of CD varies from studies (Massey et al., 2008; Reinisch et al., 2008; Mutalib et al., 2014). This pilot case series study explored the efficacy and safety of rapamycin in CD-related strictures. Overall, five patients (100%) with duodenum stricture achieved clinical response after rapamycin treatment, but none of patients with lower gastrointestinal stricture reported improvement. The present findings inspire us to precisely select the clinical application of rapamycin in CD-associated stenosis in duodenum or lesion close to duodenum.

In the present study, we found that Harvey-Bradshaw Index (HBI) could not accurately reflect the changes in the patients with CD-related gastrointestinal stricture. The evaluation based on clinical outcomes, including symptoms and tolerability, might be the better than using imaging changes. Therefore, the gastrointestinal symptoms and diet assessment were used for the clinical evaluation in these patients. We observed that clinical responses were only achieved in patients with upper gastrointestinal stricture by oral rapamycin, but not in patients with lower lesions. In this population, one male teenager with duodenum stricture who underwent repeated endoscopic dilation and percutaneous endoscopic gastrojejunostomy (PEG-J) for exclusive enteral nutrition, remained unresponsive for two years. After two months of treatment with rapamycin, he switched from enteral nutrition via tube to regular taking food by oral with normal diet, and the total score dropped from 10 at baseline to two at two months. He maintained clinical response to rapamycin for two years. Unfortunately, 10 months after withdrawal of rapamycin, gastroscopy revealed the progression of stenosis. Then he was treated with rapamycin again and has maintained response to date. Another patient with upper lesions took rapamycin continuously for only 6 months and developed rectovaginal fistula after stopping the medication. In view of these results, we suggest that rapamycin should be used as a long-term or maintenance therapy to achieve the best treatment response.

The enteral nutrition via tube can solve the nutritional needs of patients and relieve inflammatory bowel stricture in CD (Hu et al., 2014), but it seems not effective on fibrotic stenosis. Therefore, our results suggest that rapamycin may have a role in the treatment of fibrotic strictures. The present study showed a new treatment option for CD related strictures and further studies are expected to investigate the underlying mechanisms of this effect in the future.

An important question is whether rapamycin could prevent or delay surgery in this category of patients. We observed that 11/12 patients (91.7%) initially treated with rapamycin were surgery-free after a median follow-up of more than 3 years. The results may suggest that rapamycin has changed the natural history of the disease and is able to reverse strictures to some extent, which were thought to be non-reversible according to the Lemann Index in some patients with CD (Fiorino et al., 2015). In our study, the most common adverse event was mouth ulcer, and three patients discontinued the treatment due to adverse events within one month. However, adverse events occurred in patients after one month subsided spontaneously, indicating that the severity of adverse reactions may gradually decrease over time.

There were some limitations in the present study. The simple size was small, and the result might not be representative of the general population. In addition, the evaluation based on endoscopy or radiology was not performed for each patient. The benefits and risks of rapamycin for fibrotic strictures in long term should be assessed in randomized controlled trials.

In conclusion, although CD-related fibrotic stricture in upper gastrointestinal tract is rare, we had opportunity to enroll this specific population for this pilot study in China. We first time reported that the rapamycin should be effective in patients with CD-related fibrotic stricture in upper gastrointestinal tract, not those lesions in lower tract. Further case-control studies with appropriate sample and randomization are required to support the use of rapamycin in this specific subpopulation.

## Data Availability

The original contributions presented in the study are included in the article/Supplementary Material, further inquiries can be directed to the corresponding author.
